# Adolescents' Intense and Problematic Social Media Use and Their Well-Being in 29 Countries

**DOI:** 10.1016/j.jadohealth.2020.02.014

**Published:** 2020-06

**Authors:** Maartje Boer, Regina J.J.M. van den Eijnden, Meyran Boniel-Nissim, Suzy-Lai Wong, Joanna C. Inchley, Petr Badura, Wendy M. Craig, Inese Gobina, Dorota Kleszczewska, Helena J. Klanšček, Gonneke W.J.M. Stevens

**Affiliations:** a Department of Interdisciplinary Social Science, Utrecht University, Utrecht, The Netherlands; b School of Social Sciences and Humanities, Kinneret Academic College, Sea of Galilee, Israel; c Centre for Health Promotion, Public Health Agency of Canada, Ottawa, Canada; d MRC/CSO Social and Public Health Sciences Unit, University of Glasgow, Glasgow, Scotland; e School of Medicine, University of St Andrews, St Andrews, Scotland; f Faculty of Physical Culture, Palacký University, Olomouc, Czech Republic; g Department of Psychology, Queen's University, Kingston, Canada; h Department of Public Health and Epidemiology, Institute of Public Health, Riga Stradinš University, Riga, Latvia; i Institute of Mother and Child Foundation, Warsaw, Poland; j Center for Study and Development of Health, National Institute of Public Health, Ljubljana, Slovenia

**Keywords:** Social media use, Problematic social media use, Well-being, Adolescents, Cross-national research, HBSC

## Abstract

**Purpose:**

This study examined (1) whether intense and problematic social media use (SMU) were independently associated with adolescent well-being; (2) whether these associations varied by the country-level prevalence of intense and problematic SMU; and (3) whether differences in the country-level prevalence of intense and problematic SMU were related to differences in mobile Internet access.

**Methods:**

Individual-level data came from 154,981 adolescents (mean_age_ = 13.5) from 29 countries that participated in the 2017/2018 Health Behaviour in School-aged Children (HBSC) survey. Intense SMU was measured by the time spent on social media, whereas problematic SMU was defined by symptoms of addiction to social media. Mental (life satisfaction and psychological complaints), school (school satisfaction and perceived school pressure), and social (family support and friend support) well-being were assessed. Country-level data came from aggregated individual-level data and data from the Organisation for Economic Co-operation and Development (OECD) on Internet access.

**Results:**

Two-level regression analyses indicated that in countries with a lower prevalence of intense SMU, intense users reported lower levels of life satisfaction and family support and more psychological complaints than nonintense users. In contrast, in countries with a higher prevalence of intense SMU, intense users reported higher levels of family support and life satisfaction than nonintense users, and similar levels of psychological complaints. In all countries, intense users reported more friend support than nonintense users. The findings regarding problematic SMU were more consistent: In all countries, problematic users reported lower well-being on all domains than nonproblematic users. Observed differences in country-level prevalence rates of intense and problematic SMU could not be explained by mobile Internet access.

**Conclusions:**

Adolescents reporting problematic SMU are particularly at risk of lower well-being. In many countries, intense SMU may be a normative adolescent behavior that contributes positively to specific domains of their well-being.

**Implications and Contribution**

The findings from 154,981 adolescents in 29 countries showed that consistent across countries, problematic social media use, indicated by symptoms of addiction to social media, was associated with lower mental, school, and social well-being. In many countries, intense social media use, indicated by the frequency of use, related positively to specific domains of well-being.

Social media use (SMU) has become increasingly embedded in adolescents' daily lives in recent years, leading to concerns about its potential impact [1,2]. In the U.S., the percentage of adolescents who report being online almost constantly has increased from 25% to 45% between 2015 and 2018 [3]. In addition, two large-scale studies among European adolescents, conducted in 2014 and 2015, showed that the prevalence of addiction-like problematic SMU was 4.5% [4] and 9.1% [5]. Other than adolescents who merely show *intense SMU* by spending a lot of time on SMU, adolescents with *problematic SMU* typically have a diminished ability to regulate their SMU impulses, feel discomfort such as stress or anxiety when SMU is restricted, and have SMU on top of their mind constantly [6]. Research suggests that intense SMU is linked to lower mental [2,7,8], school [9], and social well-being [1] of adolescents. Moreover, problematic SMU is also associated with lower adolescent well-being [10]. However, important gaps in knowledge remain, three of which we address in this study. First, intense and problematic SMU are distinct concepts, yet correlated [[11], [12], [13]], but studies typically have not examined their associations with well-being simultaneously in one model. Therefore, it remains unclear whether intense and problematic SMU are as strongly associated with lower adolescent well-being. Second, existing research on intense and problematic SMU and their outcomes have typically used single-country data. Hence, it is not clear whether and to what extent the associations with well-being apply cross-nationally. Third, little is known about the extent to which adolescents' intense and problematic SMU differs across countries. The present study addresses these gaps by investigating independent associations of intense and problematic SMU with well-being across 29 countries.

Several mechanisms have been proposed for the negative associations of intense and problematic SMU with well-being. *Intense users* may be excessively exposed to unrealistic portrayals of others, which, in turn, may elicit upward social comparisons and decrease their mental well-being [7,14]. In addition, they may fall behind with their schoolwork because of their intense SMU, which could induce lower school well-being [9,15]. Moreover, intense users may spend less offline time with friends or family because of their intense SMU, which may have a negative impact on their social well-being [1,16]. However, there are also reasons why intense SMU may not be or only be weakly associated with low well-being. Intense SMU may be a common behavior among adolescents [3,17], as social media often play an important role in their everyday social lives [18]. Furthermore, although intense SMU indicates adolescents' time spent on SMU, it does not indicate their ability to control their SMU. Consequently, detrimental consequences of intense SMU may be limited.

In contrast, *problematic users* typically feel bad when SMU is restricted [6], which conceivably harms their mental well-being. In addition, the loss of control over and preoccupation with social media may impair their ability to regulate schoolwork responsibilities [15] and may diminish their interest in offline social activities with others [19]. As a result, problematic users may displace schoolwork and offline quality time with friends and family with SMU, which could affect their school and social well-being negatively. It, therefore, seems plausible that addiction-like problematic SMU interferes more strongly with well-being than intense SMU, yet this suggestion has rarely been investigated. The few studies that have examined adolescents' intense and problematic SMU simultaneously showed that problematic SMU, but not intense SMU, was associated with lower *mental* well-being [12,13,20]. Thus, previously found negative relationships between SMU intensity and well-being may have resulted from a confounding effect of problematic SMU.

Furthermore, the associations of intense and problematic SMU with well-being may depend on the national context. Normalization theory, which mainly has been used to explain differences in substance use between varying contexts [21,22], suggests that once risk behaviors are socially and culturally accepted by the majority of the population and have become an unremarkable feature of life [23], these behaviors may become normalized and consequently represent mainstream adolescents without problematic profiles [21]. Hence, engaging in these behaviors may not necessarily indicate lower well-being. Similarly, when intense and problematic SMU are widespread in society, these behaviors may become normalized. Consequently, when the country-level prevalence of intense or problematic SMU is high, the proposed negative associations with well-being may be low or even absent. In addition, differences in the country-level prevalence of intense and problematic SMU may be related to cross-national differences in the accessibility of mobile Internet, such as the countries' average costs and speed of mobile Internet, as adolescents typically use social media through mobile Internet devices, such as smartphones [24].

Using data from 29 countries participating in the Health Behaviour in School-aged Children (HBSC) survey (2017/2018), the present study investigated whether adolescents' intense and problematic SMU were associated with their well-being and whether these associations varied across countries. We expected that, compared with intense SMU, problematic SMU would be more strongly associated with lower mental, school, and social well-being. We also expected that associations between both types of SMU and low well-being would be weaker in countries with a higher prevalence of intense and problematic SMU. The study also investigated whether cross-national differences in the prevalence of intense and problematic SMU were related to country-level mobile Internet access. We expected that countries with more favorable mobile Internet access would report a higher prevalence of adolescent intense and problematic SMU.

## Methods

### Sample

The HBSC survey is a cross-national study that has been conducted every 4 years since 1983 to monitor the health behavior of 11, 13, and 15-year-olds across Europe, North America, and the Middle East. The present study used the 2017/2018 data, which included nationally representative data of adolescents from 47 countries/regions. Countries were excluded from the present study when individual-level data on SMU (n_countries_ = 3) or country-level data on mobile Internet accessibility were unavailable (n_countries_ = 13) or when data were not submitted by the time of current analyses (n_countries_ = 2). Adolescents who responded that the SMU questions did not apply to them were also excluded (n_individuals_ = 6,174). The analysis sample consisted of 154,981 adolescents within 29 countries/regions (51% girls; mean_age_ = 13.54; standard deviation_age_ = 1.61). Sampling methods (schools or classes as primary sampling units), data collection procedures, and questionnaires were standardized and strictly followed the HBSC international research protocol [25]. Before the survey assessments, in each country, researchers translated the English survey questions into the respective national language. Subsequently, different researchers back-translated the survey questions to English without prior knowledge of the original English survey questions. Next, language experts within the HBSC network compared the original and back-translated English survey questions. Detected inconsistencies were corrected in the national language surveys to ensure comparability of findings across different languages and cultural settings [25]. Institutional ethical consent was sought in each participating country. Participation was voluntary and anonymous, and consent was obtained from adolescents, parents, and schools.

### Individual-level measures

#### Intense SMU

Using four items adapted from the EU Kids Online Survey [26], respondents were asked how often they have online contact through social media with close friends, friends from a larger friend group, friends that they met through the Internet, and other people (e.g., parents, siblings, classmates, teachers), with responses ranging from 1 *never/almost never* to 5 *almost all the time throughout the day*, and a *do not know/does not apply* option. Respondents who answered *almost all the time throughout the day* on at least one item were classified as 1 *intense user*, and the remainder were classified as 0 *nonintense user*. The items of the scale were not expected to have high intercorrelations (e.g., adolescents with intense contact with close friends were not necessarily expected to have intense contact with friends met through the Internet). Therefore, the internal consistency of the items was not assessed [27].

#### Problematic SMU

Using the 9-item Social Media Disorder Scale [11] respondents indicated whether they, in the past year, regularly could not think of anything else but social media (preoccupation), regularly felt dissatisfied because they wanted to spend more time on social media (tolerance), often felt bad when they could not use social media (withdrawal), failed to spend less time on social media (persistence), regularly neglected other activities because of social media (displacement), regularly had arguments with others because of their SMU (problem), regularly lied to parents or friends about their time spent on social media (deception), often used social media to escape from negative feelings (escape), and had serious conflicts with parents or siblings because of their SMU (conflict). Response options were 1 *yes* and 0 *no*. Respondents who answered positively to at least six items were classified as 1 *problematic user*, and the remainder as 0 *nonproblematic user* [28]. Given the dichotomous nature of the items, internal consistency was calculated using the tetrachoric correlation matrix [29], yielding an alpha of .89.

#### Mental well-being

Two measures assessed mental well-being. Respondents rated their *life satisfaction* using Cantril's ladder [30], ranging from 0 *worst possible life* to 10 *best possible life*. The single-item nature of the measure did not allow for assessing internal consistency. However, the measure has been found to provide good test–retest reliability among adolescents [31]. A 4-item subscale from the HBSC Symptom Checklist assessed *psychological complaints* [32]. Respondents were asked how often in the last 6 months they experienced feeling low, irritable, and nervous and had difficulties falling asleep. Responses ranged from 1 *about every day* to 5 *rarely or never*. Means were computed after items were rescaled. Hence, higher mean scores indicated more psychological complaints. The internal consistency of the items was adequate (Cronbach's alpha = .75).

#### School well-being

Two measures were used. Respondents indicated their *school satisfaction*, ranging from 1 *I like it a lot* to 4 *I do not like it at all* [33]. Scores were rescaled such that high values indicated high school satisfaction. Respondents also indicated their *perceived school pressure* by rating how pressured they felt by schoolwork, ranging from 1 *not at all* to 4 *a lot* [33]. Internal consistency was not calculated, given the single-item nature of the measures. Yet, these measures have been used for many years within research using HBSC data [[33], [34], [35]].

#### Social well-being

Two 4-item subscales of the Multidimensional Scale of Perceived Social Support [36] were used to assess social well-being. The first subscale includes *family* *support* that assessed, for example, whether they can talk about problems with their family, with responses ranging from 1 *very strongly disagree* to 7 *very strongly agree*. The second subscale includes *friend support* that assessed, for example, whether they can count on friends when things go wrong. For both subscales, we calculated adolescents' mean scores. The internal consistency of both subscales was very good (Cronbach's alpha = .94 and .93).

#### Controls

The analyses were controlled for *gender*, *age* and *family affluence*. Family affluence was indicated by six items. Respondents reported the households' number of cars (0 *none*, 1 *one*, and 2 *two or more*), computers (0 *none*, 1 *one*, 2 *two*, and 3 *more than two*), and bathrooms (0 *none*, 1*one*, 2 *two*, and 3 *more than two*), whether they had their own bedroom (0 *no* and 1 *yes*), whether they had a dishwasher (0 *no* and 1 *yes*), and the number of holidays spent abroad in the past year (0 *not at all*, 1 *once*, 2 *twice*, and 3 *more than twice*). Sum-scores were transformed into proportional ranks that indicate adolescents' relative family affluence in their residential country (varying from 0 *lowest* to 1 *highest*) [37].

### Country-level measures

#### Country prevalence intense SMU

The prevalence of intense SMU was calculated as each country's proportion of respondents that were classified as intense users.

#### Country prevalence problematic SMU

The prevalence of problematic SMU was calculated as each country's proportion of respondents that were classified as problematic users.

#### Mobile Internet access

Two measures obtained from the Organisation for Economic Co-operation and Development (OECD) data were used [38]. *Costs of mobile broadband* were assessed using the countries' average price of a basket of mobile monthly usage of 300 calls and 1 gigabyte Internet in 2017. To facilitate international comparisons, prices were standardized by taking into account different price levels between countries [38]. Countries' *Internet speed* was indicated by download speed in megabits per second in 2017.

### Analysis

#### Missing data

In the analysis sample, 22.4% of respondents had missing data on at least one individual-level variable, with problematic SMU having the most missing data (9.8% of the analysis sample). To retain all respondents, missing data were imputed using multiple imputation with Mplus 8.3 [39]. Five imputations were generated using the default unrestricted “covariance” method [39]. Missing data were imputed based on available data on the individual-level study variables as well as dummy variables indicating countries to account for the nested structure of the data [40]. Iceland did not have data on Internet speed, and Lithuania did not have data on mobile broadband costs. To retain these countries, these two missing values were imputed based on available information on countries' Gross Domestic Product (GDP), number of mobile broadband subscriptions, average data usage per mobile broadband subscription [38], and countries' intense and problematic SMU prevalence.

#### Modeling

Two-level regression analyses were conducted on the imputed datasets using Mplus 8.3, with individual-level measures at the first level and country-level measures at the second level. Although the data consist of a three-level structure, where individuals were nested in schools and countries, applying three-level analyses was not feasible because then the number of parameters would exceed the number of country clusters, which does not provide model identification. In addition, to retain fewer parameters than country clusters, associations with all six well-being outcomes were examined in separate models. Models were estimated using Maximum Likelihood estimation with Robust standard errors to account for the skewed distribution of the well-being outcomes.

**Figure 1:**
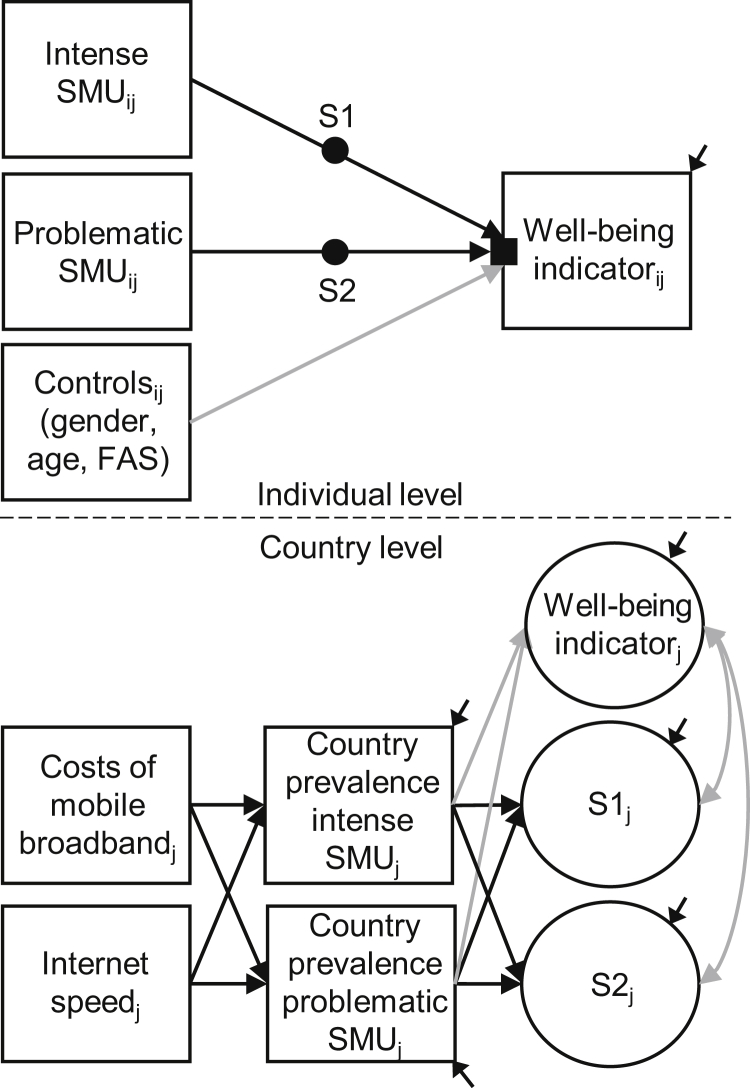
Analytical model. SMU = social media use; FAS = family affluence; Subscripts *i* and *j* denote individuals (i) in countries (j); Black circles denote random slopes (S1 and S2); Black square denotes random intercept; White squares denote observed variables; White circles denote latent variables; Grey arrows denote estimates that were added for control purposes. The analytical model was applied to all six well-being measures.

Figure 1 illustrates our analytical model, which was examined using a stepwise procedure. In our first model (denoted as M1_a_), on the individual level, we examined associations between intense and problematic SMU and life satisfaction (while controlling for gender, age, and family affluence) without any country variation, and on the country level, we tested associations between mobile Internet access and country-level prevalence of intense and problematic SMU. We extended this model with a random slope (S1) for intense SMU, which means that its association with life satisfaction was allowed to vary across countries (M1_b_). Subsequently, we added a random slope (S2) for problematic SMU (M1_c_). Next, we added two cross-level interactions that examined whether the association between intense SMU and life satisfaction varied by the country-level prevalence of intense SMU (M1_d_) and problematic SMU (M1_e_). Finally, we added two additional cross-level interactions that examined whether the association between problematic SMU and life satisfaction varied by the country-level prevalence of intense SMU (M1_f_) and problematic SMU (M1_g_). These steps were repeated for the other five well-being outcomes (M2_a-g_ to M6_a-g_).

#### Interpretations

After the stepwise analyses were conducted, for each well-being outcome, we selected the model with the best model fit for further interpretation. As a result, random slopes and cross-level interactions were only interpreted when they improved model fit. Model fit was evaluated using the Bayesian Information Criterion (BIC) and Akaike Information Criterion (AIC), where lower values indicate better model fit [41]. Furthermore, associations were interpreted using their 95% prediction intervals (PIs) [42], which indicate the range of the estimated associations across countries. Cross-level interactions were evaluated on their explained variance [43]. All continuous study variables were mean centered to facilitate interpretation of the cross-level interactions [43].

## Results

### Bivariate correlations

Table 1 shows the descriptive statistics and correlations of the individual-level and country-level variables. On the individual-level, intense and problematic SMU were correlated, with small to moderate effect size (*r* = .269). Intense SMU was associated with lower mental and school well-being, although effect sizes were small (*r* < .119). Intense SMU was associated with higher levels of friend support, with a small effect size (*r* = .117), but not with family support. Problematic SMU was correlated with lower mental, school, and social well-being, with effect sizes ranging from small (friend support: *r* = −.068) to moderate (psychological complaints: *r* = .290). At the country level, a higher prevalence of intense SMU was strongly associated with a higher prevalence of problematic SMU (*r* = .476). The cost of mobile broadband and Internet speed were not correlated with countries' intense and problematic SMU prevalence.

**Table 1:** Descriptive statistics and correlations

Individual level (n = 154,981)		Mean/proportion	SD	Minimum	Maximum	1	2	3	4	5	6	7	8
1	Intense SMU	.340				1							
2	Problematic SMU	.074				.269∗∗∗	1						
	*Mental well-being*												
3	Life satisfaction	7.639	1.846	0	10	−.042∗∗∗	−.203∗∗∗	1					
4	Psychological complaints	2.351	1.024	1	5	.119∗∗∗	.290∗∗∗	−.463∗∗∗	1				
	*School well-being*												
5	School satisfaction	2.866	.872	1	4	−.082∗∗∗	−.198∗∗∗	.295∗∗∗	−.273∗∗∗	1			
6	Perceived school pressure	2.368	.922	1	4	.101∗∗∗	.187∗∗∗	−.236∗∗∗	.333∗∗∗	−.263∗∗∗	1		
	*Social wellbeing*												
7	Family support	5.623	1.659	1	7	−.010	−.171∗∗∗	.325∗∗∗	−.246∗∗∗	.180∗∗∗	−.135∗∗∗	1	
8	Friends support	5.305	1.766	1	7	.117∗∗∗	−.068∗∗∗	.174∗∗∗	−.123∗∗∗	.141∗∗∗	−.075∗∗∗	.443∗∗∗	1
	*Controls*												
9	Female	.510				.107∗∗∗	.077∗∗∗	−.119∗∗∗	.227∗∗∗	.064∗∗∗	.124∗∗∗	−.047∗∗∗	.135∗∗∗
10	Family affluence	.502	.285	.000	.998	.033∗∗∗	−.015∗	.132∗∗∗	−.038∗∗∗	.025∗∗∗	.008	.066∗∗∗	.060∗∗∗
11	Age	13.541	1.645	10	16.5	.156∗∗∗	.094∗∗∗	−.188∗∗∗	.147∗∗∗	−.187∗∗∗	.213∗∗∗	−.123∗∗∗	−.008

Country level (n = 29)		Mean/proportion	SD	Minimum	Maximum	12	13	14	15				
12	Intense SMU prevalence	.346	.072	.174	.499	1							
13	Problematic SMU prevalence	.073	.027	.031	.142	.476∗∗∗	1						
14	Costs mobile broadband	24.341	14.792	9.849	72.506	−.024	.139	1					
15	Internet speed	15.725	3.718	7.900	23.500	−.237	−.181	−.255	1				

∗*p* < .05; ∗∗*p* < .01; ∗∗∗*p* < .001. Individual-level correlations were computed with a country cluster correction.

SD = standard deviation; SMU = social media use.

### Model selection

Table 2 shows the model fits of models (M) following a stepwise procedure. The results showed that in all models, adding random slopes for intense and problematic SMU improved model fit in terms of AIC and/or BIC (M1-6_b_ and M1-6_c_), which suggests that associations between both types of SMU and all six well-being indicators varied across countries. For life satisfaction, only the cross-level interaction between intense SMU and country-level intense SMU prevalence further improved model fit (M_1d_). The same applied to psychological complaints, although the respective cross-level interaction improved AIC, but not BIC (M_2d_). For both school well-being outcomes, models without any cross-level interaction showed the best model fit (M_3c_ and M_4c_). For both social well-being outcomes, the model with all four cross-level interactions showed the best model fit in terms of AIC, but not BIC (M_5g_ and M_6g_). For each well-being outcome, we selected the models with the best model fit for further interpretation. When AIC and BIC were inconclusive, we selected the models with the lowest AIC because these models included cross-level interactions that reduced the country variance in the investigated associations, suggesting that the respective cross-level interactions were present.

**Table 2:** Model comparisons

Model	a	b	c	d	e	f	g
	Intense + problematic SMU fixed	a + random slope intense SMU	b + random slope problematic SMU	c + intense SMU × country prevalence intense SMU	d + intense SMU × country prevalence problematic SMU	e + problematic SMU × country prevalence intense SMU	f + problematic SMU × country prevalence problematic SMU
Free parameters	18	20	22	23	24	25	26
Mental well-being							
M1. Life satisfaction							
AIC	616143.1	616075.3	616026.6	***616002.8***	616004.8	616005.3	616004.8
BIC	616322.2	616274.3	616245.6	***616231.7***	616243.6	616254.1	616263.6
u1_j_		.011	.010	*.002*	.002	.002	.002
u2_j_			.033∗∗	*.034*∗∗	.034∗∗	.031∗∗	.028∗∗
M2. Psychological complaints							
AIC	430895.9	430807.2	430730.0	***430723.3***	430723.9	430724.2	430724.2
BIC	431075.0	431006.3	**430948.9**	*430952.2*	430962.7	430973.0	430983.0
u1_j_		.005∗∗	.005∗∗	*.003*∗∗	.003∗∗	.003∗∗	.003∗∗
u2_j_			.013∗	*.013*∗	.013∗	.012∗	.011∗
School well-being							
M3. School satisfaction							
AIC	383871.5	383805.0	***383745.5***	383747.5	383747.2	383748.9	383750.8
BIC	384050.7	384004.0	***383964.4***	383976.4	383986.0	383997.7	384009.5
u1_j_		.002∗∗	*.002*∗∗	.002∗∗	.002∗∗	.002∗∗	.002∗∗
u2_j_			*.007*∗∗	.007∗∗	.007∗∗	.007∗∗	.007∗∗
M4. Perceived school pressure							
AIC	399668.6	399615.8	***399546.0***	399548.0	399549.6	399547.8	399549.3
BIC	399847.7	399814.9	***399764.9***	399776.8	399788.4	399796.6	399808.0
u1_j_		.002∗	*.002*∗	.002∗	.002∗∗	.002∗∗	.002∗∗
u2_j_			*.012*	.012	.012	.010	.010
Social wellbeing							
M5. Family support							
AIC	597242.9	597185.1	597172.6	597159.2	597158.8	597160.7	***597158.6***
BIC	597422.1	**597384.1**	597391.5	597388.1	597397.6	597409.5	*597417.4*
u1_j_		.009∗∗	.009∗∗	.004∗	.003∗	.003∗	*.003*∗
u2_j_			.012∗	.012∗	.012∗	.012∗	*.009*∗
M6. Friend support							
AIC	597587.5	597504.2	597495.2	597485.3	597484.8	597486.7	***597480.0***
BIC	597766.6	**597703.2**	597714.1	597714.1	597723.6	597735.5	*597738.8*
u1_j_		.012∗∗	.012∗∗	.007∗	.006	.006	*.006*
u2_j_			.009	.009	.009	.009	*.006*

Boldface AIC and BIC denote the lowest row values; Italics denote the models that were selected as final models for model interpretation.

SMU = social media use; *u*1_j_ = slope variance intense SMU; *u*2_j_ = slope variance problematic SMU.

### Intense SMU and well-being

**Figure 2:**
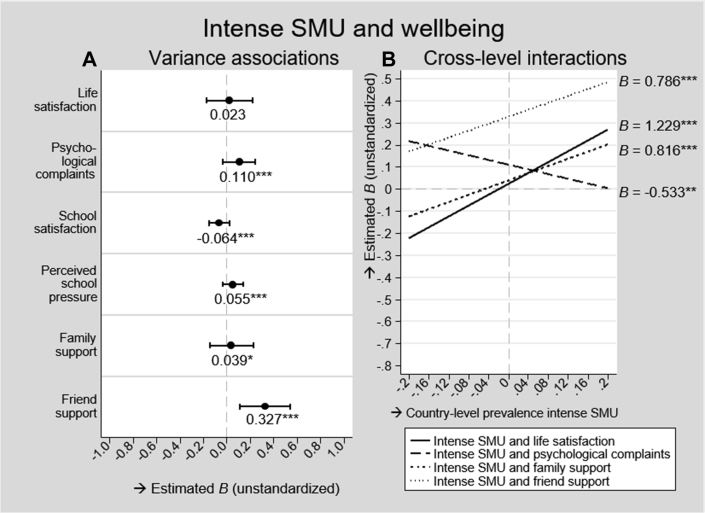
Associations between intense SMU and well-being. SMU = social media use; B = unstandardized coefficient; M = mean; ∗*p* < .05, ∗∗*p* < .01, ∗∗∗*p* < .001. Left (A): dots denote average estimated associations between intense SMU and the well-being outcomes, horizontal lines through the dots denote their 95% prediction interval. Right (B): diagonal lines represent the estimated associations of intense SMU and the well-being outcomes by country-level prevalence of intense SMU. Cross-level interactions were reported when they improved model fit and when they were significant at *p* < .05. All estimates were derived from multilevel regression models (Table A1).

Figure 2 illustrates the associations between intense SMU and well-being outcomes according to the models with the best model fit. Estimates and further details of these models can be found in the (Table A1).

#### Mental well-being

Figure 2A shows that, on average, intense SMU and life satisfaction were not related (*B* = .023; *p* = .123). However, at country-level, this association varied from negative to positive (95% PI = −.172 to .220). In countries with a higher than average prevalence of intense SMU, intense users reported higher life satisfaction than nonintense users, whereas in countries with a lower than average prevalence, intense users reported lower life satisfaction than nonintense users (*B* = 1.229; *p* < .001; Figure 2B). This cross-level interaction explained 80.0% of the country variance in this association. Adding country-level prevalence of problematic SMU as additional cross-level interaction did not improve model fit (Table 2, M1_e_).

Intense users reported more frequent psychological complaints than nonintense users (*B* = .110; *p* < .001), although this was not observed in all countries (95% PI = −.030 to .248). The higher the country-level prevalence of intense SMU, the smaller the difference in psychological complaints between intense and nonintense users, with no differences observed in the highest prevalence countries (*B* = −.533; *p* = .002; Figure 2B). Although this cross-level interaction only improved AIC, but not BIC (Table 2, M2_d_), it explained 40.0% of the country variance in this association. Adding country-level prevalence of problematic SMU as additional cross-level interaction did not improve model fit (Table 2, M2_e_).

#### School well-being

On average, intense SMU was negatively associated with school satisfaction (*B* = −.064; *p* < .001) and positively with school pressure (*B* = .055; *p* < .001), although these associations were close to zero. In some countries, the negative association with school satisfaction and the positive association with school pressure were stronger (95% PIs = −.152 to .024 and −.033 to .143, respectively). These country variances were not related to the country-level prevalence of intense and problematic SMU because models including these cross-level interactions did not show better model fit (Table 2, M3_d,e_ and M4_d,e_).

#### Social well-being

Intense and nonintense users reported about similar levels of family support on average (*B* = .039; *p* = .016). However, there was variation in this association, with intense SMU being positively related to family support in some countries and negatively related in other countries (95% PI = −.145 to .227). In countries with a high prevalence of intense SMU, intense users reported more family support than nonintense users, whereas in countries with a low prevalence, intense users reported less family support than nonintense users (*B* = .816; *p* < .001; Figure 2B). This cross-level interaction explained 55.6% of the country variance in this association. Country-level prevalence of problematic SMU did not predict any country variance in this association.

In all countries, intense users reported higher levels of friend support than nonintense users (*B* = .327; *p* < .001; 95% PI = .115–.545). The higher the country-level prevalence of intense SMU, the stronger this association was (*B* = .786; *p* < .001; Figure 2B). This cross-level interaction explained 41.7% of the country variance in this association. The results also suggested that the relationship between intense SMU and friend support was amplified by country-level prevalence of problematic SMU (*B* = 1.107; *p* = .036; not shown in Figure). However, the explanatory power of this cross-level interaction was relatively weak because it explained only 8.3% of the country variance in this relationship, and it only (marginally) improved AIC, but not BIC (Table 2, M6_e_ relative to M6_d_).

### Problematic SMU and well-being

**Figure 3:**
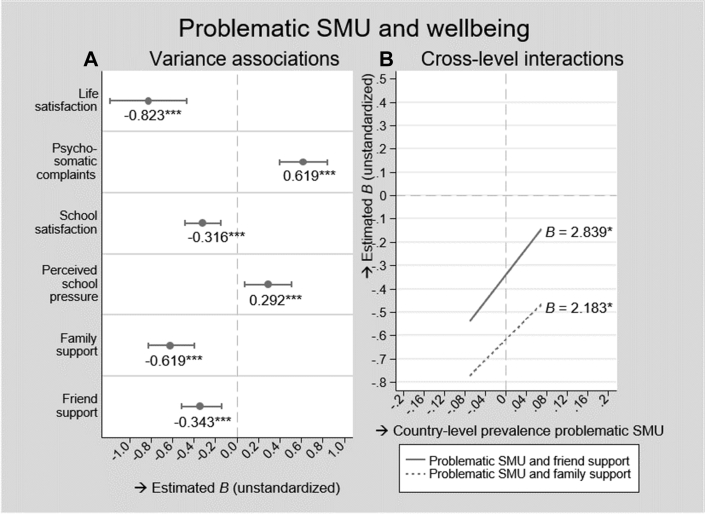
Associations between problematic SMU and well-being. SMU = social media use; B = unstandardized coefficient; M = mean; ∗*p* < .05, ∗∗*p* < .01, ∗∗∗*p* < .001. Left (A): dots denote average estimated associations between problematic SMU and the well-being outcomes, horizontal lines through the dots denote their 95% prediction interval. Right (B): diagonal lines represent the estimated associations of problematic SMU and the well-being outcomes by country-level prevalence of problematic SMU. Cross-level interactions were reported when they improved model fit and when they were significant at *p* < .05. All estimates were derived from multilevel regression models (Table A1).

Figure 3 shows the associations between problematic SMU and all well-being outcomes according to the models with the best model fits.

#### Mental well-being

Figure 3A shows that, consistent across countries, problematic users reported lower life satisfaction (*B* = −.823, *p* < .001; 95% PI = −1.179 to −.467) and more frequent psychological complaints (*B* = .619; *p* < .001; 95% PI = .396–.842) than nonproblematic users, although the strength of these associations varied across countries. This country variance was not related to the country-level prevalence of intense and problematic SMU, as adding these cross-level interactions did not improve model fit (Table 2, M1_f,g_ and M2_f,g_).

#### School well-being

Across all countries, problematic users reported lower school satisfaction (*B* = −.316; *p* < .001; 95% PI = −.480 to −.152) and higher school pressure (*B* = .292; *p* < .001; 95% PI = .077–.507). The observed country variances in the strength of these associations were not explained by country-level prevalence of intense and problematic SMU because adding these cross-level interactions did not improve model fit (Table 2, M3_f,g_ and M4_f,g_).

#### Social well-being

In all countries, problematic users reported less family support than nonproblematic users (*B* = −.619; *p* < .001; 95% PI = −.826 to −.396). The higher the country-level prevalence of problematic SMU, the weaker this association was (*B* = 2.183; *p* = .026; Figure 3B). This cross-level interaction explained 25.0% of the country variance in this association. Country-level prevalence of intense SMU did not predict any country variance in this association.

In all countries, problematic users reported lower levels of friend support than nonproblematic users (*B* = .−343 *p* < .001; 95% PI = −.516 to −.144). The higher the country-level prevalence of problematic SMU, the weaker this association was (*B* = 2.839; *p* = .011; Figure 3B), which explained 33.3% of the country variance in this association. Country-level prevalence of intense SMU did not predict any country-variance in this association.

### Cross-national differences in the prevalence of intense and problematic SMU

Table 3 shows that the prevalence of intense SMU varied from 17.35% (Switzerland) to 49.87% (Italy), whereas the prevalence of problematic SMU varied from 3.22% (the Netherlands) to 14.17% (Spain). Costs of mobile broadband and Internet speed did not explain these differences in country-level prevalence of intense SMU (*B* = .000, *p* = .628; *B* = −.005, *p* = .254) and problematic SMU (*B* = .000, *p* = .568; *B* = −.001, *p* = .338).

**Table 3:** Prevalence by country

Country	*N*	Intense SMU	Problematic SMU
Spain	4,070	38.37%	14.17%
Wales	15,456	37.26%	11.99%
Ireland	3,628	38.72%	11.99%
Italy	4,069	49.87%	10.56%
Finland	3,067	27.08%	10.16%
Greece	3,715	34.06%	9.93%
Scotland	4,916	39.31%	9.45%
Norway	3,053	39.46%	9.14%
Belgium (French)	3,695	38.32%	8.02%
Lithuania	3,685	40.90%	7.78%
England	3,306	33.91%	7.60%
Poland	5,055	43.25%	7.60%
France	8,621	36.82%	7.59%
Luxembourg	3,889	34.83%	7.37%
Canada	12,355	35.33%	6.71%
Belgium (Flanders)	4,117	43.29%	6.65%
Portugal	5,866	40.36%	5.92%
Estonia	4,622	31.42%	5.79%
Hungary	3,715	23.58%	5.39%
Latvia	4,143	25.95%	5.38%
Germany	4,126	26.15%	5.35%
Czech Republic	11,162	21.97%	5.33%
Slovenia	5,126	31.58%	5.31%
Sweden	4,006	43.10%	5.31%
Austria	4,011	33.18%	4.86%
Iceland	6,693	34.14%	4.83%
Switzerland	7,122	17.35%	4.47%
Denmark	3,113	35.04%	4.12%
Netherlands	4,579	27.53%	3.22%
Average	154,981	34.03%	7.38%

Countries were sorted on their problematic SMU prevalence.

SMU = social media use.

## Discussion

Using data from 29 countries, the present study showed that adolescents' intense SMU was positively or negatively associated with their well-being, dependent on the well-being domain and national context, whereas problematic SMU was indicative of low well-being on all investigated domains and in all countries. More specifically, in countries with a low prevalence of intense SMU, intense users reported more frequent psychological complaints, lower life satisfaction, and lower levels of family support. However, in countries with a high intense SMU prevalence, intense SMU was weakly or not associated with psychological complaints and was positively related to family support and life satisfaction. Only in some countries, intense users reported lower school satisfaction and higher school pressure than nonintense users, but this did not depend on the country-level prevalence rates of either intense or problematic SMU. Intense SMU was related to higher levels of friends support across all countries, and this association became stronger as country-level prevalence of intense SMU increased.

The findings for problematic SMU were much more consistent than for intense SMU, with lower levels of mental, school, and social well-being among problematic users in all countries, although there was country variance in the strength of these associations. This variance could not be explained by the country-level prevalence of intense and problematic SMU, except for the negative association between problematic SMU and social well-being (i.e., family support and friend support), which was stronger in countries with a lower prevalence of problematic SMU. In addition, although countries' prevalence rates of intense and problematic SMU differed substantially, these differences were not explained by the countries' mobile Internet accessibility.

By highlighting that the relationship between intense SMU and adolescent well-being depends on the well-being indicator and the national context, our results challenge the notion that intense SMU is related to lower well-being [1,2,8]. Our result support findings from systematic reviews showing that SMU can be positively and negatively associated with well-being [44,45]. In fact, given that in countries with high levels of intense SMU intense users reported higher life satisfaction and higher levels of family support than nonintense users, and that in all countries, intense users reported higher levels of friend support than nonintense users, intense SMU may often even reflect social engagement, participation, and inclusion, rather than a risk behavior.

In contrast, our findings emphasize the potential harm of problematic SMU, as problematic SMU was negatively associated with all well-being domains across all countries. This finding underlines the importance of considering intense SMU and problematic SMU as two different phenomena. The results thereby concur with previous studies showing that, although intense SMU does not necessarily indicate lower well-being, problematic SMU seems to be negatively related to multiple domains of well-being [10,12,13,20]. Hence, risks to well-being may arise, not from the time spent on SMU per se, but rather from the distinguishing features of problematic SMU, such as loss of control over SMU and preoccupation with SMU. It, therefore, seems pivotal to consider problematic SMU as a confounder when investigating the relationship between SMU intensity and well-being, as the two SMU concepts are correlated, but have different associations with adolescent well-being. Previous reports of negative associations between SMU and well-being [2,7] were therefore potentially driven by unobserved problematic SMU.

The finding that intense SMU was mainly negatively associated with well-being in countries where the prevalence of intense SMU was low, and that a low country-level prevalence of problematic SMU strengthened the negative association between problematic SMU and social well-being, is in line with other cross-national findings on adolescent well-being. For example, research suggests that the negative relationship between bullying victimization and life satisfaction is strongest in schools and countries where the prevalence of bullying victimization is low [46]. These findings suggest that normalization theory, which posits that substance use may not necessarily indicate problematic profiles in contexts where it is relatively prevalent [[21], [22], [23]], may be extended to other behaviors. That is, there may be a general pattern where specific adolescent “risk” behaviors are less indicative of problems, such as lower well-being, in contexts where many adolescents show these “risky” behaviors.

Finally, the finding that countries' mobile Internet accessibility did not predict differences in country-level prevalence of intense and problematic SMU suggests that a favorable Internet access does not increase risks related to SMU. Cross-national differences in the prevalence of intense and problematic SMU may be better explained through countries' prevailing cultural and social norms and rules regarding (social) media use, which may influence the extent to which schools and parents restrict adolescents' SMU and educate adolescents in digital literacy. However, empirical research is required to verify this possible explanation.

### Strengths and limitations

The present study has important strengths related to the number of included countries, the representative nature of the data, and the conceptual distinction between intense and problematic SMU. However, our findings should be interpreted with caution because mental, school, and social well-being were measured using either single or a few items. The use of such measures may have limited the representations of the well-being constructs, and reliability could not be established for the single-item measures. Hence, more research that replicates our study using more detailed measures of well-being is warranted. In addition, the cross-sectional design of the study does not allow for causal inferences. A reverse pattern whereby low well-being induces problematic SMU, also may be plausible [10]. Although some longitudinal studies provide evidence for a causal pathway whereby problematic SMU would negatively affect (mental) well-being [12,13], other research suggests a reverse [47] or bidirectional pathway [48]. In addition, all measures were based on self-reports that may deviate from, for example, actual time spent on SMU [49,50]. Furthermore, our measure of intense SMU was a measure of active SMU (i.e., using social media to communicate), and not of passive SMU (i.e., scrolling through profiles). A different measure of intense SMU that includes passive use may have yielded different results, as research suggests that passive use mainly decreases well-being [51,52], whereas active usage may enhance well-being [45]. Taking these limitations into account, longitudinal research on the direction of the association between (problematic) SMU and well-being, using more specific and objective measures of SMU, such as smartphone application tracking apps, are important directions for future research.

## Conclusion

Notwithstanding the previously mentioned limitations, the finding that adolescents throughout 29 countries who report problematic SMU are particularly at risk for impairments in well-being is highly relevant to current policies and guidelines for healthy SMU. Schools, family, and clinical settings are potential contexts for the detection of adolescents with problematic SMU, as well as for the implementation of support and interventions aimed at reducing the levels of problematic SMU. Additional support may be provided to adolescents reporting intense SMU in countries with a low prevalence of intense SMU because they may also be vulnerable to lower well-being.

## Figures and Tables

**Table A1:** Multilevel unstandardized results (*n*_individuals_ = 154,981, *n*_countries_ = 29)

	Mental well-being		School well-being		Social well-being	
	M1_d_ life satisfaction	M2_d_ psychological complaints	M3_c_ school satisfaction	M4_c_ school pressure	M5_g_ family support	M6_g_ friends support
	B (SE)	B (SE)	B (SE)	B (SE)	B (SE)	B (SE)
Within						
Intense SMU	.023 (.015)	.110*** (.012)	−.064*** (.010)	.055*** (.010)	.039* (.016)	.327*** (.019)
Problematic SMU	−.823*** (.038)	.619*** (.024)	−.316*** (.017)	.292*** (.021)	−.619*** (.027)	−.343*** (.026)
Cross-level						
Intense SMU × intense SMU prevalence	1.229*** (.271)	−.533** (.171)			.816*** (.234)	.786*** (.218)
Intense SMU × problematic SMU prevalence					.947 (.607)	1.107* (.526)
Problematic SMU × intense SMU prevalence					−.422 (.380)	−.460 (.338)
Problematic SMU × problematic SMU prevalence					2.183* (.977)	2.839* (1.118)

	Estimate (SE)	Estimate (SE)	Estimate (SE)	Estimate (SE)	Estimate (SE)	Estimate (SE)
Model fit						
Free parameters^a^	23	23	22	22	26	26
AIC	616002.8	430723.3	383745.5	399546.0	597158.6	597480.0
BIC	616231.7	430952.2	383964.4	399764.9	597417.4	597738.8
Random parameters						
Variance between individuals	3.117*** (.079)	.943*** (.020)	.696*** (.022)	.771*** (.023)	2.759*** (.234)	2.765*** (.167)
Variance between countries	.021*** (.006)	.021*** (.006)	.024** (.008)	.035* (.014)	.136*** (.030)	.174*** (.043)
(Residual) variance intense SMU	.002 (.001)	.003** (.001)	.002** (.001)	.002* (.001)	.003* (.002)	.006 (.003)
(Residual) variance problematic SMU	.034** (.011)	.013* (.006)	.007** (.002)	.012 (.008)	.009* (.004)	.006 (.003)
95% PI intense SMU^b^	[−.172/.220]	[−.030/.248]	[−.152/.024]	[−.033/.143]	[−.145/.227]	[.115/.545]
95% PI problematic SMU^b^	[−1.179/−.467]	[.396/.842]	[−.480/−.152]	[.077/.507]	[−.826/−.396]	[−.516/−.144]
*R*^2^ random slope intense SMU	80.00%	40.00%			55.56%	41.67% + 8.33%
*R*^2^ random slope problematic SMU					25.00%	33.33%

AIC = Akaike information criterion; BIC = Bayesian information criterion; M. = model; PI = prediction interval; *R*2 = explained variance; SE = standard error; SMU = social media use.

*p < .05; **p < .01; ***p < .001.

a Parameters not shown in the table (16): control variables female, family affluence, age, the random intercept of the respective well-being indicator, effects of the country-level prevalence of intense and problematic SMU on the random intercept, the covariances between the random intercept and the random slopes, effects of country-level costs of mobile broadband and internet speed on country-level prevalence of intense and problematic SMU, the intercepts of the country-level prevalence of intense and problematic SMU, and the residual variances of the country-level prevalence of intense and problematic SMU.

b Based on *B*'s and variance parameters of models without cross-level effects (Table 2, M1-6_c_).
